# World AIDS Day — December 1, 2018

**DOI:** 10.15585/mmwr.mm6747a1

**Published:** 2018-11-30

World AIDS Day, observed each year on December 1, draws attention to the status of the human immunodeficiency virus/acquired immunodeficiency syndrome (HIV/AIDS) epidemic worldwide. Today, approximately 36.9 million persons worldwide are living with HIV infection, including 1.8 million persons newly infected during 2017 (*1*). An estimated 940,000 persons worldwide died from AIDS-related illnesses in 2017 (*1*).

In 2015, an estimated 1.1 million persons in the United States were living with HIV infection, and 86% were aware of their infection (*2*).

Through global efforts, including the U.S. President’s Emergency Plan for AIDS Relief, for which CDC is an implementing agency, 21.7 million persons worldwide received antiretroviral therapy for HIV infection in 2017, an increase of 2.3 million persons since the end of 2016 (*1*). A report in this issue of *MMWR* (*3*) describes activities to implement the Treat All policy in India, which involves offering antiretroviral therapy to all persons with HIV infection.
